# Workplace and Healthcare Stigma in Hereditary Angioedema: Links to Anxiety and Functional Impairment

**DOI:** 10.3390/healthcare14070950

**Published:** 2026-04-04

**Authors:** Kutay Kirdok, Cenan Hepdurgun, Meryem Irem Toksoy Senturk, Atakan Citak, Sebnem Pirildar, Emine Nihal Mete Gokmen

**Affiliations:** 1 Division of Allergy and Clinical Immunology, Department of Internal Medicine, Faculty of Medicine, Ege University, 35100 İzmir, Türkiye; kirdokkutay@gmail.com (K.K.); irem.toksoy@yahoo.com (M.I.T.S.); 2 Department of Psychiatry, Faculty of Medicine, Ege University, 35100 İzmir, Türkiye; cenanhep@gmail.com (C.H.); sebnem.pirildar@ege.edu.tr (S.P.); 3 Faculty of Medicine, Ege University, 35100 İzmir, Türkiye; 01190000330@ogrenci.ege.edu.tr

**Keywords:** anxiety, hereditary angioedema, quality of life, stigma

## Abstract

**Background/Objectives**: Stigmatization is an under-recognized burden in hereditary angioedema (HAE) that may exacerbate psychosocial distress and functional impairment. Although links to adverse outcomes exist, the domain-specific pathways remain insufficiently characterized. This study investigated the impact of stigma types (workplace, healthcare, social) on anxiety, quality of life, and functional impairment, specifically testing the mediating role of disease-specific quality of life. **Methods**: This single-center, cross-sectional study included 60 adults with confirmed HAE. Participants completed the Chronic Illness Anticipated Stigma Scale (CIASS), Hospital Anxiety and Depression Scale (HADS), Angioedema Quality of Life Questionnaire (AE-QoL), and Work Productivity and Activity Impairment Questionnaire (WPAI). Hierarchical regression and mediation analyses were used to assess relationships between stigma domains, psychosocial outcomes, and activity impairment. **Results**: Female patients reported significantly higher anxiety (d = 0.85) and poorer quality of life (d = 0.77) compared to males. In hierarchical regression models, workplace stigma was a significant predictor of activity impairment (*p* = 0.002). Mediation analysis suggested an indirect association between workplace stigma and activity impairment through disease-specific quality of life (Indirect effect = 1.22; 95% CI: 0.29–3.01). **Conclusions**: Anticipated stigma in HAE appears to follow domain-specific patterns, with workplace stigma showing the strongest association with functional impairment and poorer disease-specific quality of life. Female gender emerged as an independent correlate of anxiety. These findings underscore the need for HAE management strategies that integrate psychosocial evaluation alongside biomedical control.

## 1. Introduction

Modern medical frameworks increasingly recognize that disease management must encompass not only biomedical but also psychological and social factors. Within this context, stigma is rarely a monolithic experience; rather, theoretical models distinguish between enacted stigma (actual episodes of discrimination), internalized stigma (acceptance of negative stereotypes), and anticipated stigma (the expectation of future discrimination or judgment) [1,2]. This latter form, anticipated stigma, is particularly pervasive in chronic invisible illnesses, where the constant fear of exposure and judgment can lead to poorer treatment adherence, reduced quality of life, and increased psychological distress [3,4].

Hereditary angioedema (HAE) is a rare genetic disorder characterized by recurrent, unpredictable, and disfiguring swelling episodes that can lead to significant social withdrawal and occupational disruption [5,6]. Due to the overlap of clinical features with common allergic conditions, HAE is frequently misdiagnosed, with diagnostic delays often averaging nearly two decades [7]. This prolonged ‘diagnostic odyssey’ not only engenders mistrust in the healthcare system but may also contribute to the development of internalized stigma, as patients struggle to legitimize their suffering in the absence of a confirmed diagnosis [8].

Although data on the impact of stigma in HAE remain limited, recent studies have highlighted its significance. For instance, Hews-Girard and Goodyear reported that patients frequently experience embarrassment and fear of judgment due to visible swelling, often leading them to conceal their condition [9]. Similarly, Savarese et al. demonstrated that anticipated stigma is closely linked to higher levels of anxiety and psychological distress in these patients [10].

While the existence of stigma is recognized in these preliminary reports, quantifiable data regarding its specific impact on patient outcomes remain sparse [11]. Specifically, three critical gaps exist in the current literature: (1) most studies have treated stigma as a uniform construct, failing to differentiate between domain-specific sources such as the workplace, healthcare, and social circles; (2) there is a lack of data linking these specific domains to objective functional outcomes like absenteeism; and (3) the mechanisms through which stigma affects daily functioning—whether directly or mediated by disease-specific quality of life—remain poorly understood.

**Figure 1:**
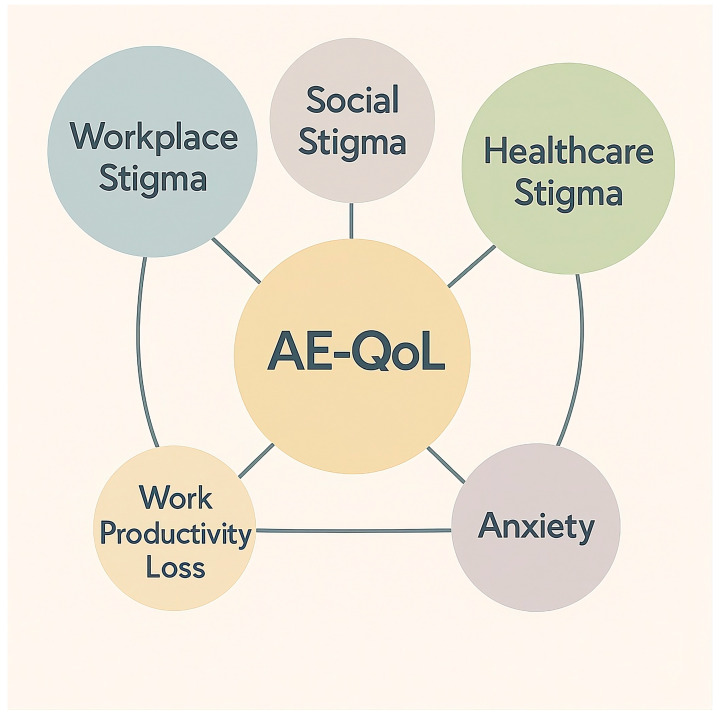
Conceptual Model: AE-QoL as Key Correlate Between Stigma and Outcomes in HAE. Note: Lines represent hypothesized pathways based on theoretical frameworks and prior literature; the cross-sectional design of this study does not permit causal inference.

To address these gaps, this study is grounded in the Chronic Illness Anticipated Stigma Framework, which posits that the expectation of stereotyping acts as a chronic stressor, driving maladaptive coping behaviors and physiological stress responses [2,12]. Applying this theoretical lens to HAE, we aimed to examine how different dimensions of stigma relate to psychological and functional outcomes. This hypothesis-driven approach was grounded in both the chronic illness stigma literature and the unique clinical features of HAE. Given that recurrent and unpredictable attacks frequently disrupt work attendance, productivity, and social functioning, workplace stigma was expected to be associated with occupational impairment [9,11]. In addition, while stigma in healthcare settings has been linked to poorer care access and quality of life in chronic illness, the diagnostic delays and substantial psychological burden commonly observed in HAE provided the rationale for examining its association with anxiety [10,12]. Finally, given the established correlations among stigma, quality of life, and psychological distress in chronic illness, disease-specific quality of life was hypothesized to mediate the relationship between stigma and functional outcomes [2,3,12]. We tested three specific hypotheses: (1) workplace stigma is associated with occupational impairment; (2) healthcare stigma is associated with anxiety; and (3) disease-specific quality of life mediates the relationship between stigma and functional outcomes. A conceptual model illustrating these hypothesized relationships is presented in Figure 1.

## 2. Materials and Methods

### 2.1. Study Design

This single-center cross-sectional study was conducted between July and November 2025. All patients were recruited and data collection was performed at the Department of Internal Medicine, Division of Allergy and Clinical Immunology, Ege University Hospital. Sixty adult patients (≥18 years) with a confirmed diagnosis of HAE were enrolled consecutively during routine follow-up visits. Diagnosis was established according to international consensus criteria [5]. Diagnosis followed established criteria for hereditary angioedema subtypes. Type I was defined by low antigenic and functional C1-INH levels, Type II by normal/elevated antigenic C1-INH with reduced function, and nC1-INH HAE by recurrent antihistamine/steroid/anti-IgE-refractory angioedema with normal C1-INH parameters plus either a pathogenic variant or a positive family history. Patients with acquired angioedema or conditions impairing questionnaire reliability were excluded.

The study was approved by the local institutional ethics committee, and written informed consent was obtained from all participants. The study was conducted in accordance with the principles of the Declaration of Helsinki.

### 2.2. Data Collection

Data were collected face-to-face by a physician (K.K.) during outpatient clinic visits using a structured case report form. To ensure clinical accuracy, the implementation and interpretation of psychiatric measures were supervised by two consultant psychiatrists (C.H. and S.P.). Sociodemographic variables included age, gender, education level, marital status, family structure, and place of residence. Clinical data included HAE type, disease duration, attack frequency and localization, current treatment modality, and access to healthcare services. Access to on-demand treatment was recorded as a patient-reported variable indicating whether the participant experienced difficulty obtaining on-demand treatment when needed; the specific reasons for limited access were not systematically recorded. In addition, family history of diagnostic delay was recorded as a yes/no variable indicating whether the participant reported delayed diagnosis in at least one affected family member; the duration of delay and the specific family member were not systematically recorded. Patient diagnostic delay, by contrast, was analyzed as a continuous variable in years. At the time of data collection, the prophylactic therapies available and reimbursed in the Turkish healthcare system for HAE included plasma-derived C1-INH concentrate and danazol; donidalorsen was available only through an expanded access program, whereas lanadelumab and berotralstat were not approved or reimbursed in Turkey during the study period. For on-demand treatment, the available options in our setting included icatibant and plasma-derived C1-INH concentrate. Laboratory parameters (C1-INH levels/function and C4) represent basal values obtained during routine visits. Psychosocial measures are described below. Gender was determined by self-report; no participants in this sample identified outside the female/male binary. Data on race or ethnicity were not collected as the study population is ethnically homogeneous.

### 2.3. Measures

Anticipated stigma: assessed using the validated Turkish version of the Chronic Illness Anticipated Stigma Scale (CIASS) [13]. The 12-item instrument evaluates anticipated stigma from family/friends, coworkers/employers, and healthcare providers on a 5-point Likert scale, where higher scores indicate greater stigma. The workplace stigma subscale was administered to participants who had worked at least once in their lifetime, regardless of whether they were currently employed at the time of the study.

Anxiety and depression: evaluated with the Turkish version of the Hospital Anxiety and Depression Scale (HADS), which contains 14 items scored from 0 to 3 (7 for anxiety and 7 for depression). Although the original version uses a cut-off score of ≥11 for each subscale, previous Turkish validation studies have recommended a cut-off of ≥10 for anxiety and ≥7 for depression [14].

Quality of life: Health-related quality of life was measured using the Turkish version of the Angioedema Quality of Life Questionnaire (AE-QoL). This disease-specific questionnaire has been culturally adapted and linguistically validated for Turkish-speaking individuals with HAE. Total and domain scores were calculated according to the standardized scoring method, with higher scores reflecting worse quality of life [15].

Work productivity: assessed using the Turkish version of the Work Productivity and Activity Impairment Questionnaire: General Health (WPAI-GH) [16]. This instrument has been validated in Turkish and assesses absenteeism, presenteeism, overall work impairment, and activity impairment within the previous seven days. Activity impairment was calculated from item 6 and was available for 53 participants. In contrast, absenteeism, presenteeism, and overall work impairment were calculated only for employed participants with complete work-related WPAI data (n = 25 of 28 employed participants).

### 2.4. Statistical Analysis

All analyses were performed using IBM SPSS Statistics version (IBM Corp., Armonk, NY, USA; v26.0). Data normality was assessed using the Kolmogorov–Smirnov test, histogram inspection, and skewness–kurtosis values. Descriptive statistics were reported as mean ± standard deviation (SD) for continuous variables and frequency (percentage) for categorical variables. Two separate diagnostic delay variables were assessed: (1) patient diagnostic delay (continuous), defined as the time interval in years between symptom onset and confirmed diagnosis, which was used in correlation and regression analyses; and (2) family history of diagnostic delay (categorical, Yes/No), defined as whether the participant reported delayed diagnosis in at least one affected family member, which was used in group comparison analyses. These are distinct variables and are labeled accordingly in Table 1.

Group comparisons (e.g., sex, treatment access) were conducted using independent samples t-tests. To quantify the magnitude of differences between groups, Cohen’s d effect sizes were calculated and interpreted as small (0.2), medium (0.5), and large (0.8). Pearson correlation coefficients were used to assess bivariate associations. Given the exploratory nature of the study and the large number of pairwise correlations, the Benjamini–Hochberg false discovery rate (FDR) correction was applied to the bivariate correlation matrix. Correlations surviving FDR correction at q < 0.05 are indicated separately, whereas nominally significant correlations that did not survive correction should be interpreted as hypothesis-generating rather than confirmatory.

Because missing data varied across instruments, the analytic sample size differed by outcome. Correlations involving absenteeism, presenteeism, and overall work impairment were restricted to employed participants with complete work-related WPAI data (n = 25), whereas correlations involving activity impairment were based on participants with available item 6 data (n = 53). Workplace stigma analyses were based on available CIASS workplace subscale responses from participants with prior work experience (n = 56). Anxiety and activity impairment were selected as the principal dependent variables because they represent the psychological and functional dimensions of patient burden, respectively. Activity impairment was preferred over work-specific WPAI outcomes for multivariable and mediation analyses because it is applicable regardless of employment status and was therefore available for a larger proportion of the cohort.

To examine the independent contributions of stigma domains to psychosocial and occupational outcomes, hierarchical multiple regression analyses were conducted. Covariates for each regression model were selected a priori based on established clinical and theoretical associations with the respective outcome variables, rather than on the basis of bivariate significance in the present dataset [5,9]. In the first step, demographic or clinical covariates were entered depending on the outcome variable (age and sex for anxiety; sex and abdominal attack frequency for activity impairment). The stigma subscale entered in the second step was determined according to the predefined hypotheses (healthcare stigma for anxiety; workplace stigma for activity impairment). AE-QoL total score was entered in the third step as a prespecified variable to assess whether quality of life accounted for the variance initially attributed to stigma. Model fit was evaluated using the change in the coefficient of determination and F-statistics.

To test the hypothesis that disease-specific quality of life mediates the relationship between workplace stigma and activity impairment, a mediation analysis was performed using the PROCESS macro (v4.3, Andrew F. Hayes; available online: https://www.processmacro.org, accessed on 29 November 2025 for SPSS.) In this analysis, activity impairment was used as the functional outcome because it reflects overall daily functioning across the cohort, unlike work-specific WPAI outcomes, which were restricted to employed participants. The significance of the indirect effect was determined using 5000 bootstrap samples to generate 95% bias-corrected confidence intervals (CIs). An indirect effect was considered statistically significant if the 95% CI did not include zero. Given the cross-sectional design and the limited complete-case sample available for the mediation model, this analysis was considered exploratory and intended to examine whether the observed associations were compatible with a potential indirect pathway rather than to establish causal ordering. Statistical significance was set at *p* < 0.05.

The sample size of 60 was determined by the number of confirmed HAE patients available at our center during the study period. While no formal a priori power analysis was conducted, the rarity of the disease constrains feasible enrollment. For the primary regression models with four predictors, n = 60 is sufficient (≥0.80) to detect medium-to-large effects (f^2^ ≥ 0.20) at α = 0.05 [17].

## 3. Results

### 3.1. Participant Characteristics

A total of 60 patients with a confirmed diagnosis of HAE were included. The mean age was 40.8 ± 15.7 years, and 68.3% of the participants were female. The majority were married (60%) and had a university or higher education level (45%). Most patients (78.3%) resided in urban areas. Regarding their clinical care, although all patients were recruited and assessed at our university hospital, 93.3% (n = 56) are primarily followed up in university hospital clinics for their routine HAE management, while 6.7% (n = 4) also utilize state hospital services.

Regarding clinical phenotypes, the majority of patients had type I HAE (83.3%), followed by type II HAE (10%) and HAE with normal C1-inhibitor (6.7%). The mean baseline C4 level was 6.02 ± 2.67, the mean C1 inhibitor level was 4.35 ± 2.80, and the mean C1 inhibitor function was 17.29 ± 10.90. The mean diagnostic delay was 16.4 ± 14.7 years. Nearly half of the participants (46.7%) were actively employed, and 48.3% were receiving long-term prophylactic treatment. Of the 37 patients reporting ≥24 attacks per year, 29 (78.4%) were receiving prophylaxis, consistent with the occurrence of breakthrough attacks despite treatment in real-world HAE management. Detailed sociodemographic and clinical characteristics are presented in Table 1.

### 3.2. Scale Scores and Descriptive Results

The total anticipated stigma score was 26.6 ± 8.4 (n = 56). Among the subscales, workplace stigma had the highest scores (11.7 ± 4.3, n = 56), followed by healthcare stigma (7.6 ± 3.2, n = 60) and social stigma (7.2 ± 4.1, n = 60).

HADS-Anxiety and HADS-Depression scores were 8.6 ± 3.9 and 6.3 ± 3.8, respectively (n = 59). The AE-QoL total score was 46.6 ± 14.2 (n = 39).

Functional outcomes were analyzed based on employment status and data availability. For the employed subgroup (n = 25), the overall work impairment was 55.6% and the absenteeism rate was 20.3%. In the broader sample, the daily activity impairment was calculated as 47.5% (n = 53). Descriptive statistics for all scales and subdomains are presented in Table 2.

### 3.3. Correlation Analyses

Bivariate correlation analyses demonstrated that workplace stigma was associated primarily with occupational and quality-of-life measures, whereas healthcare stigma showed closer associations with psychological distress and selected quality-of-life outcomes. Full results of the correlation matrix, including FDR-corrected significance, are presented in Table 3.

Among patients with HAE due to C1-inhibitor deficiency, no significant associations were found between biochemical parameters (C1 inhibitor level, C1 inhibitor function, or C4 level) and psychosocial outcomes, including stigma, anxiety, depression, quality of life, or work impairment (all *p* > 0.05).

### 3.4. Group Comparisons

**Figure 2:**
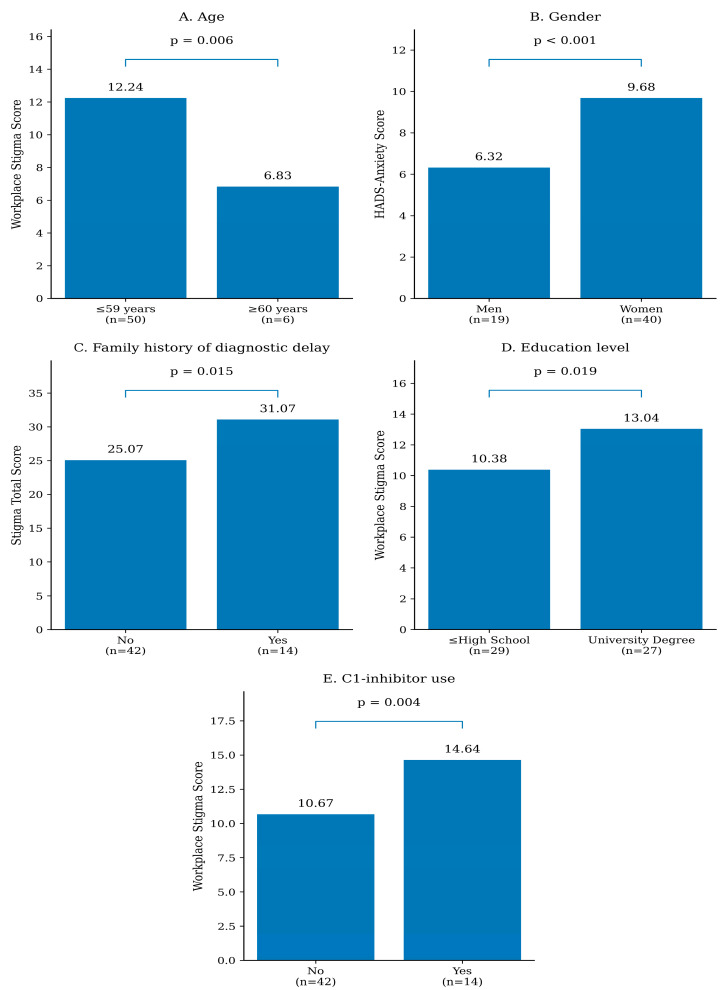
Variations in Psychosocial Burden and Work Impairment Across Patient Subgroups. Note: Subgroup sizes reflect the number of participants with available data for the specific outcome shown in each panel (**A**–**E**).

Group comparisons were conducted to examine whether sociodemographic and clinical variables were associated with stigma and psychosocial outcomes. Age was dichotomized at 60 years, consistent with the threshold commonly used in HAE literature to identify elderly patients with potentially different disease management profiles [18]. All statistically significant group differences are shown in Figure 2.

Specifically, female patients reported significantly higher levels of anxiety compared to males (9.56 ± 4.0 vs. 6.45 ± 2.6; *p* = 0.003), with a large effect size (Cohen’s d = 0.85). Similarly, females exhibited significantly poorer disease-specific quality of life (higher AE-QoL scores) compared to males (49.47 ± 14.1 vs. 39.00 ± 11.4; *p* = 0.040, Cohen’s d = 0.77).

As a descriptive observation, the four patients reporting limited access to on-demand treatment had higher healthcare stigma scores than those with access (12.50 ± 3.5 vs. 7.28 ± 2.9). Given the very small subgroup size, this finding should be interpreted with caution.

Other sociodemographic and clinical variables, including place of residence, having a general family history of HAE (as distinct from diagnostic delay), and general prophylactic treatment status, were also tested and found to have no significant association (all *p* > 0.05).

### 3.5. Hierarchical Regression Analyses

Two hierarchical regression models were constructed to examine the determinants of anxiety and activity impairment.

Model 1: Predictors of Anxiety

In the prediction of HADS-Anxiety scores (Table 4; n = 39), demographic variables (age and sex) entered in Step 1 explained a significant portion of the variance (R2 = 0.299, *p* = 0.002), with female sex being the strongest predictor (beta = −0.499, *p* = 0.001). The addition of healthcare stigma in Step 2 did not explain significant additional variance (Delta R2 = 0.025, *p* = 0.257). Similarly, the addition of AE-QoL in Step 3 yielded only a marginal increase in explanatory power (Delta R2 = 0.057, *p* = 0.084).

Model 2: Predictors of Activity Impairment

In the prediction of activity impairment (Table 5), clinical variables (abdominal attack frequency and sex) in Step 1 explained 8.7% of the variance (*p* = 0.222). In Step 2, the addition of workplace stigma resulted in a significant increase in explanatory power (Delta R2 = 0.244, *p* = 0.002), with workplace stigma acting as a strong independent predictor (beta = 0.506, *p* = 0.002). In Step 3, adding AE-QoL total score further increased the model’s explanatory power by 22.4% (Delta R2 = 0.224, *p* < 0.001), bringing the total explained variance to 55.5%. Upon the addition of AE-QoL, the beta coefficient for workplace stigma decreased from 0.506 to 0.325 but remained significant (*p* = 0.019), suggesting a partial mediation effect.

### 3.6. Mediation Analysis

A mediation analysis was performed to examine whether the observed association between workplace stigma and activity impairment was compatible with an indirect pathway through AE-QoL (n = 36). The model controlled for sex and abdominal attack frequency. Workplace stigma was significantly associated with the mediator, AE-QoL (a path: B = 1.18, SE = 0.56, *p* = 0.043). In turn, AE-QoL was significantly associated with activity impairment (b path: B = 1.04, SE = 0.26, *p* < 0.001). The direct effect of workplace stigma on activity impairment remained significant (c’ path: B = 2.19, SE = 0.88, *p* = 0.019). The indirect effect through AE-QoL was statistically significant (effect = 1.22, BootSE = 0.63; 95% bias-corrected bootstrap CI [0.29, 3.01]; completely standardized indirect effect = 0.18). The ratio of indirect to total effect (P_M) was 0.36, suggesting that approximately 36% of the total association between workplace stigma and activity impairment may operate through quality of life. Given the cross-sectional design and the limited complete-case sample available for the mediation model, this analysis was considered exploratory and intended to examine whether the observed associations were compatible with a potential indirect pathway rather than to establish causal ordering.

## 4. Discussion

In this study, our three a priori hypotheses were partially supported. First, workplace stigma emerged as the stigma domain most clearly related to occupational functioning. Second, healthcare stigma was more closely associated with psychological distress, particularly anxiety, than with work-related functioning. Third, although anticipated stigma showed significant bivariate associations with psychosocial and occupational outcomes, these associations were substantially attenuated after inclusion of AE-QoL in multivariable analyses, and mediation analysis suggested that disease-specific quality of life may partly explain the relationship between workplace stigma and activity impairment.

These findings were also reflected in the bivariate analyses, where anticipated stigma showed significant associations with anxiety and work-related outcomes. Importantly, the CIASS captures anticipated stigma, that is, the expectation of future judgment or discrimination, rather than enacted stigma or broader perceived societal devaluation. However, when disease-specific quality of life was examined in regression analyses, the independent contribution of stigma was significantly attenuated. Instead, day-to-day well-being, as captured by the AE-QoL total score, showed the strongest independent associations with both psychological distress and work impairment. To further investigate these associations, our mediation analysis suggested that the relationship between workplace stigma and activity impairment was compatible with both a direct association (*p* = 0.018) and an indirect association through deterioration in quality of life (Indirect effect = 1.22; 95% CI: 0.29–3.01). This pattern is consistent with reports from other chronic conditions, where higher anticipated stigma has been associated with greater emotional distress and reduced work performance [19,20,21]. Given that the confidence interval was wide, indicating limited precision, and since the mediation model was based on a limited complete-case sample, this finding should be interpreted cautiously and viewed as exploratory rather than confirmatory. While our mediation model suggests a pattern consistent with an indirect association between workplace stigma and reduced productivity through quality of life, it is important to note that the cross-sectional design precludes definitive causal inferences. Bidirectional relationships are possible; for instance, patients with higher functional impairment might anticipate greater stigma.

Consistent with our first and second hypotheses, stigma in HAE was not a single, uniform experience; rather, its relevance varied across social and clinical settings. In this cohort, workplace stigma was most prominent and showed moderate associations with absenteeism and reduced productivity. Similar workplace-related patterns have been described in other chronic diseases, particularly when stigma originates from colleagues or supervisors [22,23]. In contrast, stigma related to healthcare interactions was more closely associated with psychological distress, particularly anxiety symptoms, than with work-related functioning. These patterns may reflect patients’ experiences within the healthcare system, influenced in part by historically prolonged diagnostic pathways, although the underlying mechanisms remain unclear. Comparable findings have been reported in other chronic illnesses, where perceived disapproval or judgment has been associated with greater emotional distress and lower quality of life [24]. Unexpectedly, the social stigma subscale was not significantly associated with any psychosocial or occupational outcome. This null finding may reflect the high prevalence of familial HAE in our cohort (71.7%), which could foster understanding and reduce stigmatization within close social circles.

Several clinical factors appeared to influence the functional impact of the disease. Specifically, abdominal attack frequency showed positive associations with both absenteeism and activity impairment. This is likely due to the debilitating nature of abdominal attacks, which are characterized by severe, cramping pain and often nausea/vomiting. These symptoms can be physically incapacitating, forcing patients to remain at home and preventing them from attending work or social activities, thereby directly increasing absenteeism rates. The absence of similar associations for other attack sites further highlights that abdominal episodes may impose a uniquely heavy burden on daily functioning [25].

Experiences within the healthcare system also appeared to be relevant, consistent with earlier studies emphasizing the importance of treatment access and care-related challenges [26,27]. Patients reporting limited access to on-demand treatment had higher anxiety scores and higher healthcare stigma scores. However, this subgroup was very small (n = 4), and the corresponding effect size estimate is likely unstable; these findings should therefore be interpreted with considerable caution and regarded as descriptive and hypothesis-generating rather than confirmatory.

Several sociodemographic factors showed meaningful variation in workplace-related stigma. Younger patients and those with higher educational attainment reported higher scores, which may reflect heightened sensitivity to professional expectations or career-related pressures in these groups. In addition, patients receiving C1-inhibitor prophylaxis reported greater workplace stigma. It is important to note that in our country, intravenous C1-inhibitor prophylaxis is administered exclusively in healthcare facilities and is not available for home self-administration. Consequently, the requirement for frequent hospital visits during working hours increases the visibility of the disease and necessitates repeated requests for time off, likely intensifying anticipated stigma in the workplace.

Interestingly, patients who reported a history of diagnostic delay in at least one affected family member had significantly higher total stigma scores. One possible explanation is that shared family experiences related to delayed diagnosis may influence how stigma is anticipated within affected families. This interpretation is consistent with previous observations regarding the shared psychosocial burden borne by families and caregivers in HAE [11,28]. However, this explanation remains speculative and cannot be established from our cross-sectional data.

Our findings also suggest that the timing of key disease milestones may have psychosocial relevance. Earlier symptom onset was associated with higher stigma scores, indicating that long-term exposure to HAE-related limitations may shape stigmatization experiences [8]. Conversely, a later age at diagnosis correlated with higher depressive symptoms, a pattern consistent with the psychological impact of longer patient diagnostic delay described in the previous HAE literature [29].

The finding that female patients exhibited significantly higher anxiety levels is in line with previous HAE reports showing a greater psychological burden among women. In our cohort, this gender disparity was substantial, with female sex showing large effect sizes for both anxiety (d = 0.85) and poorer quality of life (d = 0.77). This highlights the need for gender-sensitive approaches in clinical care [30,31].

One of the most notable findings of this study was the apparent disconnect between objective clinical markers and psychosocial outcomes. No significant associations were observed between biochemical parameters and any psychosocial measures. Furthermore, total annual attack frequency was not correlated with stigma, anxiety, or work impairment. These results indicate that neither laboratory indices nor the simple count of attacks adequately reflect the patients’ psychological burden or functional difficulties. A patient with few but unpredictable attacks may experience as much anxiety or stigma as one with frequent attacks. This underscores a critical gap: traditional metrics of disease severity often fail to capture the lived experience of HAE [22,32,33].

This study extends previous work by suggesting that anticipated stigma in HAE is not a single, uniform construct. Rather, its subdomains—most notably workplace and healthcare stigma—demonstrate different patterns of association with psychological and functional outcomes. In our analyses, disease-specific quality of life showed the strongest independent associations, with significant links to both anxiety and work-related limitations. Collectively, these findings emphasize the clinical importance of incorporating patient-reported experiences into routine care. Such an approach may provide a more accurate understanding of the challenges faced by individuals with HAE than reliance on laboratory data alone.

Our findings suggest that interventions should be domain-specific: workplace stigma may require occupational support and employer education, while healthcare stigma may warrant greater attention to communication and patient experience within clinical care. Finally, the dissociation between biomarkers and patient burden supports the integration of patient-reported outcome measures into routine monitoring.

This study has several methodological limitations. First, its cross-sectional design precludes causal interpretation of the associations observed among stigma, quality of life, and psychosocial outcomes. Second, the single-center setting and relatively small sample size (n = 60)—although expected for a rare condition such as HAE—may limit the generalizability of the findings. In particular, the predominance of middle-aged adults in our sample may restrict the applicability of the results to pediatric and elderly HAE populations. Moreover, the study was conducted in a healthcare system where intravenous prophylaxis is hospital-based; therefore, workplace stigma findings may differ in countries where home-based self-administration is available. Third, although the mediation analysis yielded a statistically significant indirect effect, the complete-case sample for this model was limited and the confidence interval was wide, indicating limited precision; accordingly, this finding should be interpreted as exploratory and hypothesis-generating. Simulation-based studies have shown that substantially larger samples may be required for adequately powered mediation analyses [34]. Fourth, all psychosocial variables were assessed using self-report instruments, which may introduce biases such as social desirability or common method variance. Some contextual variables were recorded in limited detail: family history of diagnostic delay was captured only as a yes/no variable, and limited access to on-demand treatment was assessed as a patient-reported variable without systematic documentation of the underlying reasons. Finally, biochemical parameters were measured at a single time point, and the use of a generic instrument such as the WPAI-GH may not fully capture work-related impairments specific to HAE.

## 5. Conclusions

This study provides new evidence on the psychosocial experiences of patients with HAE. Anticipated stigma showed significant associations with anxiety and work-related outcomes; however, multivariable analyses suggested that disease-specific quality of life may represent an important explanatory pathway in these associations. These findings suggest that higher anticipated stigma is associated with poorer work-related functioning and psychological well-being, and that these associations may be partly accounted for by worse disease-specific quality of life, although causal inferences cannot be drawn from this cross-sectional design.

The domain-specific nature of stigma was evident: workplace stigma showed the strongest association with occupational impairment, whereas healthcare-related stigma was more closely linked to psychological distress. In contrast, biochemical markers were not meaningfully associated with psychological or functional outcomes, revealing a disconnect between biological severity and lived experience.

Overall, these findings highlight the multidimensional nature of patient burden in HAE and underscore the necessity of integrating psychosocial assessments into routine clinical care. Future multicenter, longitudinal studies are warranted to validate these observations and to inform targeted interventions—such as improving treatment access and workplace support—aimed at ameliorating outcomes in this rare disease population.

## Figures and Tables

**Table 1:** Sociodemographic and Clinical Characteristics of Patients.

**Part A: Categorical Variables**			
**Demographic Characteristics**		**n**	**%**
Gender			
	Woman	41	68.3
	Man	19	31.7
Age			
	59 years and below	53	88.3
	60 years and above	7	11.7
Education level			
	High school or below	33	55.0
	University (Bachelor’s degree) and above	27	45.0
Marital status			
	Single	24	40.0
	Married	36	60.0
Family history of hae			
	Yes	43	71.7
	No	17	28.3
Place of residence			
	Village-district	13	21.7
	Province	47	78.3
Family diagnostic delay history			
	Yes	15	25.0
	No	45	75.0
Employment status (n = 47) ^a^			
	Employment	28	59.6
	No employment	19	40.4
HAE type			
	1	50	83.3
	2	6	10.0
	nC1-INH	4	6.7
Primary institution for routine HAE care			
	University hospital	56	93.3
	State hospital	4	6.7
Acute attack frequency in the past year (n = 59) ^b^			
	0–23 attacks	22	37.3
	24 or more attacks	37	62.7
Long-term prophylaxis			
	Yes	29	48.3
	No	31	51.7
Prophylactic agent among LTP users ^c^			
C1 esterase inhibitor		16 (55.2%)	
Danazol		9 (31.0%)	
Donidalorsen		4 (13.8%)	
**Part B: Continuous Variables**			
**Variable**	**n**	**Mean ± SD**	**Median (IQR)**
Age (years)	60	40.8 ± 15.7	39.0 (29.0–50.3)
Symptom onset age (years)	60	11.1 ± 9.8	8.5 (5.0–14.3)
Patient diagnostic delay (years) ^d^	60	16.4 ± 14.7	13.5 (3.8–25.0)
Acute attack frequency (past year)	59	58.7 ± 62.5	40.0 (20.0–71.0)
Annual abdominal attack frequency	59	27.7 ± 30.4	16.0 (5.0–45.0)

HAE: Hereditary angioedema; nC1-INH: Normal C1 inhibitor level; SD: Standard Deviation; LTP: Long-term prophylaxis. ^a^ 13 participants had missing employment data. ^b^ Data regarding acute attack frequency was missing for one participant (n = 59). ^c^ Percentages for prophylactic agents were calculated among patients receiving long-term prophylaxis (n = 29). ^d^ Patient’s diagnostic delay (years) is defined as the interval between symptom onset age and age at confirmed diagnosis. This is a separate variable from the family diagnostic delay history reported in Part A.

**Table 2:** Descriptive Statistics for Psychosocial Scales and Work Productivity Measures.

Variable	Possible Range	n	Mean ± SD	Median	Min	Max
CIASS Total Score	12–60	56	26.57 ± 8.40	26.00	12.0	43.0
Social Stigma	4–20	60	7.18 ± 4.09	5.50	4.0	19.0
Workplace Stigma	4–20	56	11.66 ± 4.30	12.00	4.0	20.0
Healthcare Stigma	4–20	60	7.55 ± 3.21	7.00	4.0	16.0
HADS Anxiety	0–21	59	8.59 ± 3.94	8.00	1.0	20.0
HADS Depression	0–21	59	6.32 ± 3.81	6.00	1.0	15.0
AE-QoL Total	0–100	39	46.62 ± 14.19	46.00	19.0	87.0
AE-QoL Functioning	0–100	39	44.26 ± 17.90	44.00	0.0	69.0
AE-QoL Fatigue/Mood	0–100	39	44.10 ± 19.22	50.00	10.0	95.0
AE-QoL Fears/Shame	0–100	39	52.74 ± 20.18	50.00	17.0	92.0
AE-QoL Nutrition	0–100	39	39.31 ± 27.40	25.00	0.0	88.0
WPAI Absenteeism (%)	0–100	25	20.28 ± 28.71	0.00	0.0	100.0
WPAI Presenteeism (%)	0–100	25	48.40 ± 29.54	50.00	0.0	90.0
WPAI Overall Work Imp. (%)	0–100	25	55.65 ± 33.41	40.00	0.0	100.0
WPAI Activity Impairment (%)	0–100	53	47.55 ± 29.54	50.00	0.0	100.0

Note. CIASS = Chronic Illness Anticipated Stigma Scale; HADS = Hospital Anxiety and Depression Scale; AE-QoL = Angioedema Quality of Life Questionnaire; Imp.= Impairment; WPAI = Work Productivity and Activity Impairment Questionnaire. WPAI work-specific domains (absenteeism, presenteeism, overall work impairment) are restricted to employed participants. Higher scores indicate greater stigma, more symptoms, lower quality of life, and greater impairment, respectively.

**Table 3:** Bivariate Correlations Among Psychosocial Measures (Lower Triangle).

	1	2	3	4	5	6	7	8	9	10	11	12	13	14	15
1. Stigma Total	—	—	—	—	—	—	—	—	—	—	—	—	—	—	—
2. Social Stigma	0.75 **†	—	—	—	—	—	—	—	—	—	—	—	—	—	—
3. Workplace Stigma	0.81 **†	0.40 **†	—	—	—	—	—	—	—	—	—	—	—	—	—
4. Healthcare Stigma	0.60 **†	0.26 *	0.31 *	—	—	—	—	—	—	—	—	—	—	—	—
5. HADS Anxiety	0.25	0.06	0.26	0.28 *	—	—	—	—	—	—	—	—	—	—	—
6. HADS Depression	0.03	0.03	−0.01	0.08	0.62 **†	—	—	—	—	—	—	—	—	—	—
7. AE-QoL Total	0.28	0.03	0.36 *	0.27	0.47 **†	0.31	—	—	—	—	—	—	—	—	—
8. AE-QoL Functioning	0.27	−0.12	0.39 *	0.33 *	0.24	0.06	0.66 **†	—	—	—	—	—	—	—	—
9. AE-QoL Fatigue	0.04	−0.09	0.05	0.30	0.37 *	0.45 **†	0.76 **†	0.47 **†	—	—	—	—	—	—	—
10. AE-QoL Fears/Shame	0.26	0.07	0.28	0.13	0.28	0.10	0.71 **†	0.17	0.27	—	—	—	—	—	—
11. AE-QoL Nutrition	0.21	0.16	0.34 *	−0.10	0.44 **†	0.28	0.61 **†	0.35 *	0.35 *	0.25	—	—	—	—	—
12. Absenteeism	0.10	−0.31	0.39	0.10	0.47	0.36	0.77 **†	0.78 **†	0.67 *	0.58	0.35	—	—	—	—
13. Presenteeism	0.13	0.00	0.18	0.23	0.15	0.06	0.55 *†	0.65 **†	0.55 *†	0.29	0.05	0.60 *	—	—	—
14. Overall Work Imp.	0.00	−0.23	0.24	0.08	0.20	0.20	0.78 **†	0.90 **†	0.82 **†	0.39	0.31	0.77 **†	0.93 **†	—	—
15. Activity Impairment	0.26	0.04	0.37 **†	0.14	0.40 **†	0.23	0.57 **†	0.51 **†	0.30	0.50 **†	0.24	0.58 *	0.89 **†	0.82 **†	—

*Note.* * *p* < 0.05; ** *p* < 0.01; † survives Benjamini–Hochberg FDR correction at q < 0.05. Pearson correlations are reported except for correlations involving social stigma score and absenteeism, for which Spearman rank correlations were used due to non-normal distributions. Correlations involving absenteeism (12), presenteeism (13), and overall work impairment (14) are based on employed participants only. 1 = Stigma Total; 2 = Social Stigma; 3 = Workplace Stigma; 4 = Healthcare Stigma; 5 = HADS Anxiety; 6 = HADS Depression; 7 = AE-QoL Total; 8 = AE-QoL Functioning; 9 = AE-QoL Fatigue; 10 = AE-QoL Fears/Shame; 11 = AE-QoL Nutrition; 12 = Absenteeism; 13 = Presenteeism; 14 = Overall Work Impairment; 15 = Activity Impairment.

**Table 4:** Hierarchical Regression Analysis Predicting HADS Anxiety (n = 39).

Variable	B	SE	β	t	*p*	R^2^	ΔR^2^
Step 1						0.299	0.299
Age	−0.057	0.033	−0.241	−1.718	0.095		
Sex (male)	−4.181	1.128	−0.521	−3.705	<0.001 ***		
Step 2						0.342	0.043
Age	−0.048	0.033	−0.202	−1.437	0.160		
Sex (male)	−4.046	1.112	−0.504	−3.637	<0.001 ***		
Healthcare stigma	0.220	0.145	0.211	1.510	0.140		
Step 3						0.408	0.065
Age	−0.043	0.032	−0.181	−1.332	0.192		
Sex (male)	−3.337	1.132	−0.415	−2.948	0.006 **		
Healthcare stigma	0.150	0.145	0.144	1.040	0.306		
AE-QoL total	0.070	0.036	0.281	1.938	0.061		

*Note.* ** *p* < 0.01; *** *p* < 0.001. Sex coded as 0 = female, 1 = male. Covariates (age, sex) were selected a priori based on established clinical associations.

**Table 5:** Hierarchical Regression Analysis Predicting Activity Impairment (n = 36).

Variable	B	SE	β	t	*p*	R^2^	ΔR^2^
Step 1						0.087	0.087
Sex (male)	9.468	10.305	0.153	0.919	0.365		
Abdominal attacks	0.248	0.157	0.264	1.583	0.123		
Step 2						0.331	0.244
Sex (male)	14.032	9.054	0.227	1.550	0.131		
Abdominal attacks	0.179	0.138	0.191	1.300	0.203		
Workplace stigma	3.412	0.998	0.506	3.420	0.002 **		
Step 3						0.555	0.224
Sex (male)	23.127	7.846	0.374	2.948	0.006 **		
Abdominal attacks	0.131	0.115	0.139	1.139	0.264		
Workplace stigma	2.193	0.882	0.325	2.486	0.018 *		
AE-QoL total	1.037	0.262	0.542	3.952	<0.001 ***		

*Note.* * *p* < 0.05; ** *p* < 0.01; *** *p* < 0.001. Sex coded as 0 = female, 1 = male. Covariates (sex, abdominal attack frequency) were selected a priori based on clinical rationale.

## Data Availability

The data presented in this study are available on request from the corresponding author due to privacy and ethical restrictions.

## Abbreviations

The following abbreviations are used in this manuscript:

AE-QoL	Angioedema Quality of Life Questionnaire
C1-INH	C1 Esterase Inhibitor
C4	Complement Component 4
CIASS	Chronic Illness Anticipated Stigma Scale
HADS	Hospital Anxiety and Depression Scale
HADS-A	HADS Anxiety Subscale
HADS-D	HADS Depression Subscale
HAE	Hereditary Angioedema
SPSS	Statistical Package for the Social Sciences
IgE	Immunoglobulin E
nC1-INH HAE	Hereditary Angioedema with Normal C1 Inhibitor
*p*	*p*-Value
QoL	Quality of Life
r	Correlation Coefficient
SD	Standard Deviation
WPAI-GH	Work Productivity and Activity Impairment Questionnaire: General Health
